# Immunohistochemical expression of sulfhydryl oxidase (QSOX1) in pediatric medulloblastomas

**DOI:** 10.1186/s13000-015-0268-2

**Published:** 2015-04-24

**Authors:** Ana Cristina Lira Sobral, Victor Moreschi Neto, Gabriela Traiano, Ana Paula Percicote, Elizabeth Schneider Gugelmin, Cleber Machado de Souza, Lia Nakao, Luiz Fernando Bleggi Torres, Lucia de Noronha

**Affiliations:** School of Medicine, Pontifical Catholic University of Paraná, Curitiba, Brazil; Department of Basic Pathology and Department of Medical Pathology, Federal University of Paraná, Curitiba, Brazil; Head of the Anatomic Pathology Service at the Pequeno Príncipe Hospital, Curitiba, Brazil; School of Health and Biosciences, Pontifical Catholic University of Paraná, Curitiba, Brazil

**Keywords:** Immunohistochemical expression, Sulfhydryl oxidase, QSOX1, Pediatric medulloblastomas

## Abstract

**Background:**

Medulloblastoma is a malignant, invasive embryonal tumor of the cerebellum and accounts for 20% of intracranial tumors in children. QSOX1, whose functions include formation of disulphide bridges, which are needed for correct protein folding and stability, formation of the extracellular matrix, regulation of the redox status and cell cycle control, appears to be involved in apoptosis in pathological states such as cancer. Thus, the aim of this study was to investigate the immunohistochemical expression of QSOX1 in medulloblastomas and nonneoplastic cerebellum.

**Methods:**

Histology blocks of pediatric medulloblastomas were separated and two representative areas of the tumors and non-neoplastic cerebellum samples were used to construct tissue microarrays (TMAs) that were stained with an anti-QSOX1 antibody, and the slides were read using image analysis software.

**Results:**

QSOX1 immunoexpression was observed in the non-neoplastic cerebellum samples and the medulloblastoma samples. There was no statistically significant relationship between QSOX1 immunopositivity in the medulloblastoma samples and the clinical and pathological variables.

**Conclusions:**

Although QSOX1 did not prove useful for stratifying patients into risk groups, tumor cells and the fibrillar extracellular matrix were positive for this marker, indicating that this enzyme may be involved in the pathogenesis of medulloblastoma.

**Virtual Slides:**

The virtual slide(s) for this article can be found here:

http://www.diagnosticpathology.diagnomx.eu/vs/1822040654139436

## Background

Medulloblastoma is a malignant embryonal tumor of the nervous system. It has histological features reminiscent of a neuroepithelial tumor and consists of small round blue cells embedded in a glial extracellular matrix (ECM), or neuropile.

During the last decade, new discoveries in molecular biology have shown that stratification of embryonal tumors into risk groups based only on histological and clinical criteria is of limited. Specific genetic mutations should also be investigated, as these are very often characteristic of more aggressive tumors [1]. Furthermore, in spite of the new surgical and chemotherapeutic techniques continually proposed for embryonal tumors, the prognosis for medulloblastoma remains poor. Patients initially considered low-risk may relapse and behave like high-risk patients. In light of this, various studies of embryonal tumors have also used new biomarkers that stratify these tumors into more accurate prognostic groups.

QSOX1 belongs to a family of proteins initially described in the reproductive system of male rodents and involved in the formation of disulphide bridges, which are needed for the correct folding and functioning of proteins responsible for the formation of the ECM, growth control, apoptosis and cell signaling [2]. It plays an important role in the induction of apoptosis in response to stress-inducing events, in mesenchymal cell differentiation in embryos and in ECM maturation, especially in nerve tissue. In view of this, as medulloblastomas are made up of embryonic cells and maturation may be related to a better prognosis for these tumors, expression of QSOX1 in medulloblastomas would be expected to be related to better biological behavior. In addition, expression of QSOX1 may be related to better response to chemotherapy because of its role in the induction of apoptosis in response to stress-inducing events [3,4]. This study reports immunohistochemical expression of QSOX1 in medulloblastomas and human cerebellum, the former for the first time.

## Methods

### Patients and tissue specimens

Twenty nine medulloblastoma samples from the anatomic pathology service at the Pequeno Príncipe Children’s Hospital taken from patients with their diagnosis confirmed between 1998 and 2009 were used. The study was approved by the research ethics committees at the Pontifical Catholic University of Paraná and the Pequeno Príncipe Hospital (number 0518–07).

### Immunohistochemistry

Histological slides stained with hematoxylin-eosin were reviewed, and the samples classified according to their histological type. Two representative samples of each tumor (fifty-eight cores) and twelve samples/cores of non-neoplastic cerebellum were selected and used to prepare five tissue microarrays (TMAs) [5].

Histological sections were cut from these blocks and incubated with mouse anti-human-QSOX1 recombinant monoclonal antibody produced at the Federal University of Paraná (UFPR) [3,6]. Advance^TM^ HRP Dako® was used as the secondary antibody. The immune reactions were developed by adding DAB chromogen-substrate solution (Dako®) to the slides.

### Evaluation of immunoreactivity

Immunohistochemical expression of QSOX1 was evaluated using the morphometric and color functions in Image Proplus® image processing and analysis software running on a Dell® computer coupled to a Dino Eye® camera and an Olympus BX40 microscope. A sample of normal cerebellum was used as a reference against which the immunohistochemical expression of the anti-QSOX1 antibody could be evaluated. Immunopositive areas in a 400x high-power field of this cerebellum sample were input in the Image Proplus® software, which stores them as a mask. The mean immunopositive area per high-power field in the medulloblastomas was calculated using four images for each sample taken with a 40x objective. The image from a normal cerebellum (the mask) was superimposed on each of these images using the Image Proplus® software, which identifies those areas where the staining is similar to that in the mask, i.e., immunopositive areas, and calculates the mean total immunopositive area per high-power field in mm^2^.

The following clinical data were obtained from the twenty nine patients’ medical records: age at diagnosis; risk group; type of surgical resection (whether complete or not); whether there were tumor relapses after surgery; how long after surgery these relapses occurred; presence or otherwise of metastases at diagnosis; use or otherwise of post-surgery adjuvant chemotherapy; whether the patient died after the treatment; and how long after surgery death occurred. Patients were followed up for between 2 and 12 years.

The variables were tested for normality with the Kolmogorov-Smirnov test. The non-parametric Mann–Whitney test was used to compare quantitative variables between the various groups. The significance level was set at p < 0.05. The data were analyzed with Statistica 8.0.

## Results

Mean age at diagnosis of the 29 patients in the study was 4.8 years (1 month to 15 years). Nineteen patients were three years or older, and twenty were high risk, i.e., they were under three years of age and/or had a residual tumor after surgery and/or metastases were present at diagnosis [1]. All the patients underwent surgery as recommended in the literature. After the surgery, 15 still had residual lesions. None of the patients presented with metastases at diagnosis. Post-surgery relapses were observed in nine patients and occurred 1.7 years after surgery on average. Twenty-six patients needed post-surgery chemotherapy. Three of the 29 patients died; the average time between diagnosis and death was 32.7 days (Table 1).

**Table 1:** **Descriptive statistics for the clinical variables in the group of patients with medulloblastoma**

**Clinical course**	**Number of patients (29)**	**Percentage**
Disease-free	26	89.7
Death	3	10.3
Age at diagnosis		
<3 years	10	34.5
≥3 years	19	65.5
Total resection		
Yes	14	48.3
No	15	51.7
Post-surgery relapse		
Yes	9	31.0
No	20	69.0
Metastases at diagnosis		
Yes	0	0
No	29	100
Post-surgery ct		
Yes	26	89.7
No	3	10.3
Risk group		
Low	9	31.0
High	20	69.0

ct = chemotherapy.

Twenty-eight (96.6%) of the medulloblastomas were classic and one (3.4%) desmoplastic. Mean QSOX1 immunoexpression in all the medulloblastomas was 0.0135 mm^2^. Median QSOX1 immunoexpression was analyzed for correlation with the clinical variables (age; whether under or over three years of age; high or low risk; total or incomplete resection; presence of post-surgery relapses; use of post-surgery chemotherapy; and death). The results are shown in Table 2.

**Table 2:** **Relationship between mean immunohistochemical expression of QSOX1 in medulloblastomas and total surgical resection, post-surgery relapse, death, age at diagnosis and risk group**

**Clinical and pathological variables**		QSOX1*		
		**Mean**	**Median**	p value**
Death	Yes	0.013	0.012	
	No	0.012	0.013	0.635
Age at diagnosis	<3 years	0.013	0.013	
	≥3 years	0.013	0.012	0.131
Total surgical resection	Yes	0.013	0.012	
	No	0.012	0.012	0.981
Post-surgery relapse	Yes	0.012	0.012	
	No	0.013	0.012	0.481
Risk group	Low	0.014	0.012	
	High	0.013	0.013	0.738

*QSOX1-immunopositive area (mm^2^); **Mann–Whitney test (p value < 0.05).

**Figure 1:**
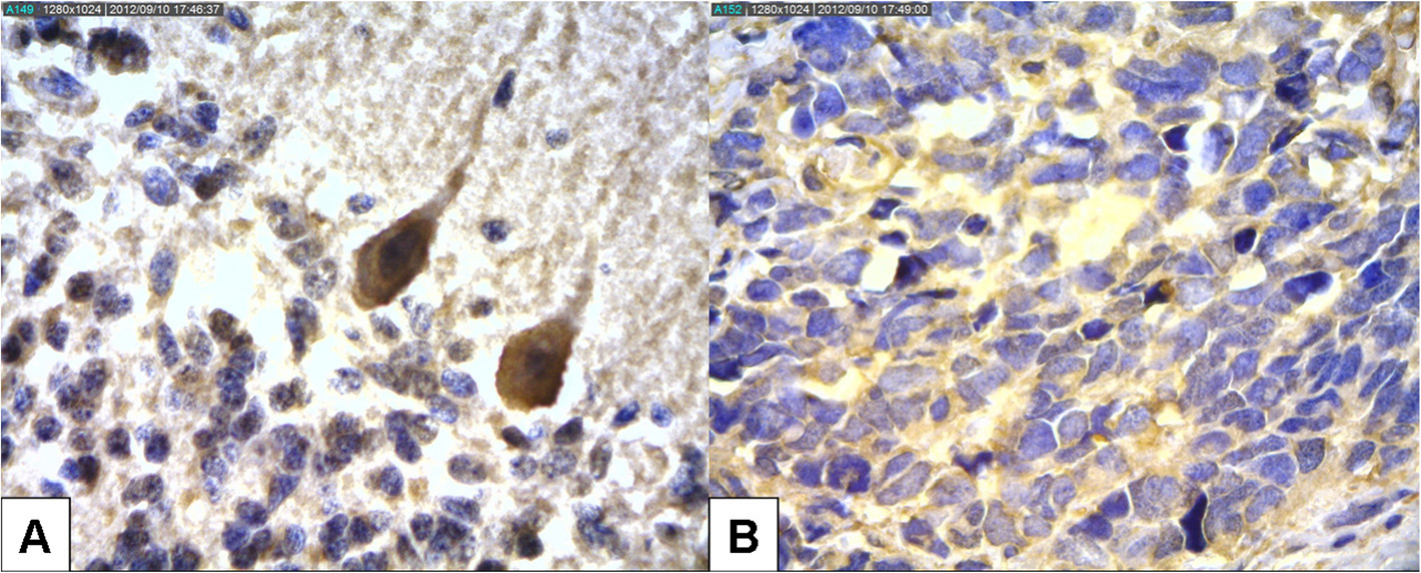
Immunoexpression of QSOX1 in tumor-free cerebellum and in a medulloblastoma. **A**. Immunopositivity of Purkinje cells X400. **B**. Immunopositivity of the medulloblastoma X200.

Analysis of normal cerebellar tissue revealed positive staining for QSOX1 in the fibrillar matrix and Purkinje cells and an absence of staining in granule cells (Figure 1).

In samples of classic medulloblastomas (N = 28), the fibrillar matrix (neuropile) exhibited intense, diffuse staining. Similar staining was also occasionally observed in the cytoplasm of neoplastic cells, particularly in the perinuclear region. QSOX1 immunohistochemical staining in desmoplastic medulloblastomas (N = 1) was similar to that observed in classic medulloblastomas. Although positivity in the matrix in the former was greater, this can be explained by the greater abundance of matrix in this type of tumor.

## Discussions

Although the role of sulfhydryl oxidases has not been fully elucidated, an understanding of their potential substrates and functions can be gained from their cellular distribution [7]. The extracellular location of QSOX1 suggests that it may be involved in cell signaling or ECM remodeling [8]. QSOX1 is also found in the intracellular environment in various organelles, especially in the endoplasmic reticulum and Golgi complex [8-10].

The sulfhydryl oxidase QSOX1 is expressed in various human tissues, such as those from the myocardium, placenta, stomach, lung, liver, brain, kidney, skeletal muscle and pancreas [3,11].

QSOX1 has been found in the Golgi complex, suggesting that one of its main functions is to act in the extracellular compartment [8,12-14]. Although in humans QSOX1 has been described in both the Golgi complex and endoplasmic reticulum, the fact that it has been found in the Golgi complex in the central nervous system of mice suggests that it is involved in maintenance of the ECM [14].

This oxidase is involved in the formation of disulphide bridges in certain proteins that are responsible for the formation of the ECM [8,12,13,15], which in turn plays a role in growth control mechanisms. In addition, as QSOX1 has a role in ECM-cell interactions, it may induce apoptosis [11].

The interest in studying QSOX1 expression in the central nervous system arose because of the role this enzyme plays in maintaining the redox state, which may be related to neuronal death [8,12,13]. Expression of QSOX1 has been detected in the neuronal Golgi apparatus in mice, particularly in the cerebellum, where it was observed in cerebellar neurons in deep nuclei and the cerebellar cortex and was most intense in dentate, globose, emboliform and fastigial nuclei [16]. In the same study, Purkinje cells and granule cells in the cerebellar cortex did not stain, unlike the Golgi cells in the granular layer, particularly those in contact with Purkinje cells [16].

The existence of QSOX1 in neuronal bodies that secrete neuropeptides may indicate that this enzyme also has intracellular functions. QSOX1 has also been found in mouse fetuses, highlighting the role it plays during the development of the nervous system, when it may be involved in neuronal migration and maturation [16].

Studies of QSOX1 expression during embryonic development of the mouse nervous system found that Purkinje cells express QSOX1 temporarily during the neuronal maturation phase of the postnatal period, when dendritic processes grow and remodel, synapses form and there is interaction with glial processes [16].

The immunopositivity observed in the fibrillar matrix and Purkinje cell bodies in non-neoplastic cerebellum samples from children with medulloblastoma may reflect the role of this protein in the developing and maturing cerebellum. Positive staining for QSOX1 in the fibrillar matrix and cytoplasm of medulloblastoma cells suggests that this enzyme is involved in the formation of the ECM in these tumors, while the positive staining observed in the perinuclear region of medulloblastoma cells may be related to the fact that QSOX1 is found in the Golgi complex.

The present study has limitations that need to be clearly discussed. The fewer number of blocks with medulloblastoma could be considered a limitation. However, this number eventually becomes significant because the number of patients with problem is small. The original observation of the impact of presence of QSOX-1 on patient in the present study can provide information for future studies.

## Conclusions

QSOX1 is detected in human cerebellar tissue and medulloblastoma samples; however, determining expression of this enzyme does not help in the identification of risk groups as it does not appear to be associated with a worse prognosis. The immunohistochemical expression of this enzyme reported here reinforces the findings of previous studies using animal models and highlights the need for further studies into the role of this enzyme in the pathogenesis of medulloblastoma.

## Abbreviations

ECM: Glial extracellular matrix
TMA: Tissue microarray
